# Advancing Fintech through a transdisciplinary approach

**DOI:** 10.1016/j.isci.2023.107694

**Published:** 2023-09-07

**Authors:** Debora Jackson, Kwamie Dunbar, Joseph Sarkis, Robert Sarnie

**Affiliations:** 1 Worcester Polytechnic Institute, Worcester, MA 01609, USA

## Abstract

The term “Fintech” is a portmanteau of “financial technology.” Fintech is an emerging field that can be advanced through interdisciplinary collaboration, contributing to the fourth industrial revolution, known as Industry 4.0 (Doherty & Stephens, 2023). It is used to augment, disrupt, or enhance traditional financial services (Thakor, 2023). However, there is no universally agreed-upon definition for Fintech, as it varies depending on the characteristics of Fintech companies and the sectors, they operate in. The advancement of technology, particularly in the Fintech industry, has sparked extensive debates on the opportunities and challenges for businesses, consumers, and the labor force. *The introduction of new Fintech technologies is expected to disrupt educational systems at all levels*, *necessitating the cultivation of fresh talent*, *which*, *in turn*, *requires an immediate and proactive response from higher education institutions and the business community*. This collaboration between higher education and industry partners requires re-evaluation in order to equip the workforce with the skills needed through skilling, reskilling, and upskilling.

**Figure:**
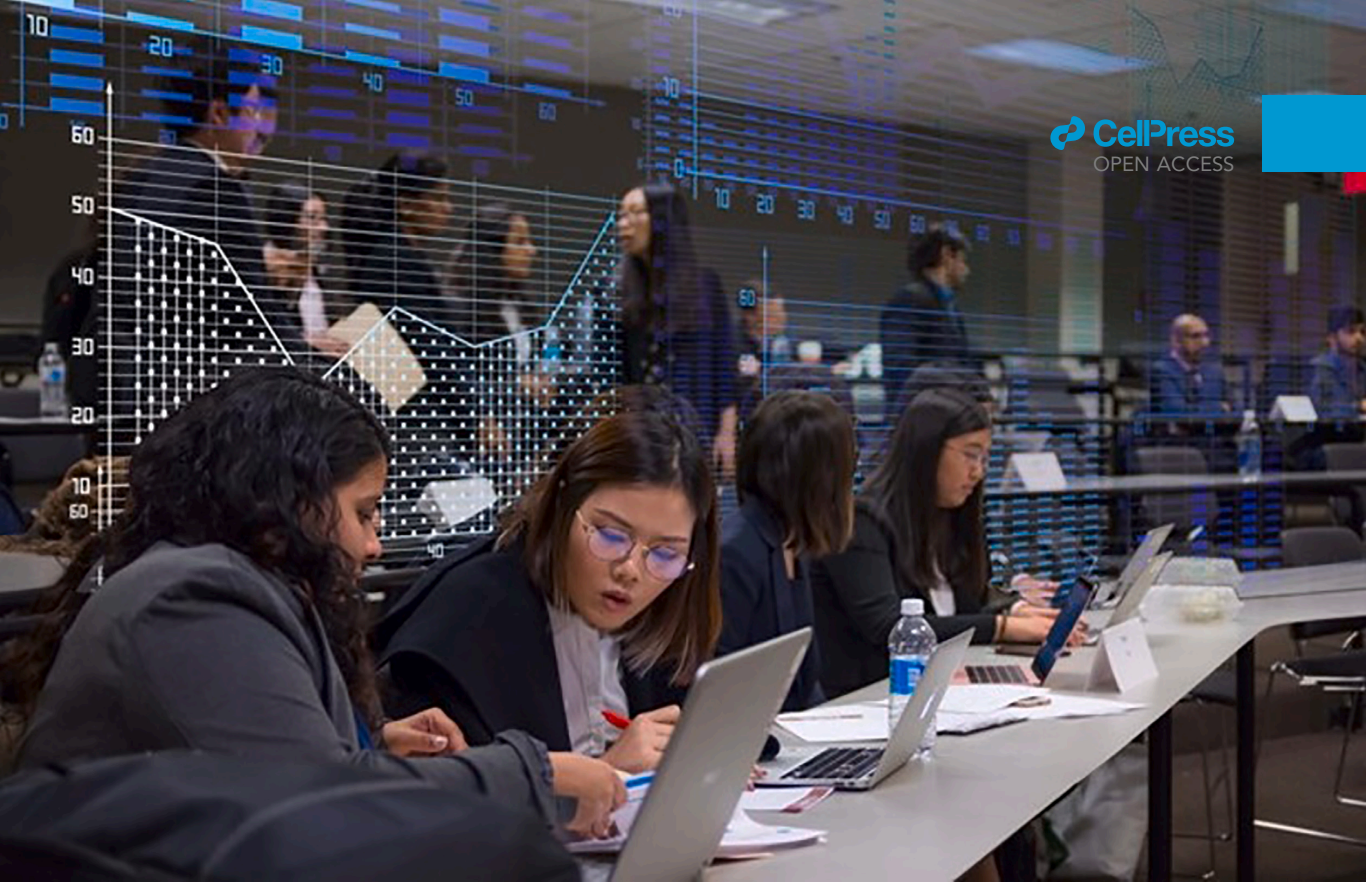
Fintech has the potential to disrupt traditional educational programs and training In this Backstory, the emerging program focused on Fintech at Worcester Polytechnic Institute is discussed with several key stakeholders and put in context with the interdisciplinary field.

The introduction of new Fintech technologies is expected to disrupt educational systems at all levels, necessitating the cultivation of fresh talent, which, in turn, requires an immediate and proactive response from higher education institutions and the business community.

Transdisciplinary cooperation in Fintech education fosters professionals who possess a comprehensive understanding of various fields and can address real-world challenges.

WBS’ distinctive competence in its cross-disciplinary experiential learning, Major Qualifying Projects (MQP) (administered by our Fintech MQP, FinTech Project Center), positions our students and faculty for the interdisciplinary requirements of new Fintech programs.

The key factors that stimulate interdisciplinary research include fostering a culture of collaboration and encouraging the exchange of ideas among researchers from different disciplines.

For example, we have management, finance, and computer science scholars and students attempting to address the question of how blockchain can be used to manage sensor data that may have social good implications, while developing a business model for this technology and the company partner.

## Main text

The term “Fintech” is a portmanteau of “financial technology.” Fintech is an emerging field that can be advanced through interdisciplinary collaboration, contributing to the fourth industrial revolution, known as Industry 4.0 (Doherty & Stephens, 2023).^1^ It is used to augment, disrupt, or enhance traditional financial services (Thakor, 2023).^2^ However, there is no universally agreed-upon definition for Fintech, as it varies depending on the characteristics of Fintech companies and the sectors, they operate in. The advancement of technology, particularly in the Fintech industry, has sparked extensive debates on the opportunities and challenges for businesses, consumers, and the labor force. *The introduction of new Fintech technologies is expected to disrupt educational systems at all levels*, *necessitating the cultivation of fresh talent*, *which*, *in turn*, *requires an immediate and proactive response from higher education institutions and the business community*. This collaboration between higher education and industry partners requires re-evaluation in order to equip the workforce with the skills needed through skilling, reskilling, and upskilling. (Caciatori et al., 2020; Brown, 2020).^3^^,^^4^

This backstory is a unique look of Fintech’s potential disruptive impact on higher education. It aims to provide an understanding of higher education’s response to this transformative field’s skills demand, thus aligning academic curricula with the dynamic industrial needs. The insights presented in this backstory—which are based on extensive research and interviews with industry experts, Fintech startups, and educators—will provide valuable information for businesses and policymakers in the Fintech sector and the overall financial services industry.

In the modern knowledge economy, knowledge-intensive innovation and entrepreneurship (KIE) are considered the most significant forms of entrepreneurship, because they involve the transformation of technology and ideas into innovation. KIE plays a vital role in fostering economic growth. For instance, in Massachusetts, Fintech startups, such as SaaSWorks, play a pivotal role in converting technological expertise into economic knowledge, leading to higher levels of innovation performance through knowledge commercialization. Entrepreneurship, especially in the high-tech sector, is a key driver of innovation (Chung, Jung, & Lee, 2022).^5^

To achieve broad success in Fintech, higher education institutions must adapt and support new emerging transformative technologies (e.g., the artificial intelligence revolution is now gripping the financial industry) (Shaw & Gani, 2023),^6^ while maintaining a strong theoretical foundation (Mercorio & Mezzanzanica, 2019).^7^ Traditional higher education structures with specialized disciplines hinder interdisciplinary collaboration; however, advancements in technology and the need for innovation call for programs that foster cooperation (Liao et al., 2017).^8^ Overcoming disciplinary barriers and collaborating with external stakeholders, such as financial institutions, regulators, communities, and civil society, is essential for the development and application of Fintech (Doherty & Stephens, 2023).^1^

Fintech is not limited to developed countries and high-tech business programs; it can benefit a wide range of institutions. That is, Fintech can range in support from smallholder family farms in developing countries to researchers in developed nations and premier global scientific institutions. While Fintech is not a solution to all problems, when studied and developed with a focus on integrating natural sciences, engineering, mathematics, social sciences, business, and external stakeholders, it can serve as an enabler.

Universities often encounter situations in which newly developed technologies require practical application and monetization efforts, which are crucial for their long-term adoption and diffusion. For example, collaborations between computer scientists, information technology experts, user experience researchers, and medical professionals aim to provide incentives for using healthcare information devices. Fintech provides insights into and mechanisms for these incentive systems. Econsult Solutions Inc. conducted an independent study to measure the economic impact of 59 private colleges and universities that comprise the Association of Independent Colleges and Universities in Massachusetts (Retelle et al., 2023).^9^ This association includes eight private higher education institutions located in Central Massachusetts. The study revealed that Worcester Polytechnic Institute (WPI), through research and innovation, generated noteworthy economic effects such as the establishment of startups, the acquisition of patents, and the formation of license agreements. Currently, WPI holds a stake in 22 companies that collectively employ 335 individuals and have raised more than $1 billion in funding.

Considering the significant societal, social, and economic implications that Fintech can potentially have, it is crucial for firms and higher education institutions to collaborate with a goal of accurately identifying the required skill sets. In this backstory, we consider the impact of Fintech on society and the recently established programs in Fintech at WPI which may serve as a model of an interdisciplinary program in the area.

### Fintech’s societal impact

Fintech has the potential to address broad societal challenges, such as the United Nations’ sustainable development goals (SDGs). For example, Fintech can alleviate poverty by improving our understanding of social structures and providing inclusive financial systems. Fintech can foster the advancement of societal welfare through the provision of financial services to marginalized communities. This characteristic enables financial inclusion to play a pivotal role in diminishing poverty, stimulating economic progress, and realizing the United Nations’ SDGs. Fintech also relates to other SDGs, including sustainable consumption and production (SDG 12), technological innovation, and economic development (SDG 8 and 9, respectively). It can also address focused environmental concerns such as climate change (SDG 13) through carbon trading and offset mechanisms.

Consequently, the question facing the private sector, government, and higher education is—how can we support the attainment of these goals? According to the 2021 National Biennial Survey of unbanked and underbanked households conducted by the Federal Deposit Insurance Corporation (FDIC), which is dedicated to enhancing Americans’ access to safe, secure, and affordable banking services, around 4.5% of U.S. households, equivalent to approximately 5.9 million households, were identified as “unbanked.” “Today, almost 80% of adults in the world are still either underbanked or unbanked (Boston Consulting Group, 2023).”^10^ The FDIC’s findings also revealed that the prevalence of unbanked households varied significantly across different demographic groups and has been a historical pattern. For instance, higher unbanked rates were observed among lower-income households, households with lower levels of education, Black households, Hispanic households, working-age households with disabilities, and single-mother households—some of our most vulnerable and underrepresented communities.

By leveraging technological innovation, Fintech can offer accessible and affordable financial services to underserved communities, reducing barriers that prevent individuals from accessing traditional banking services. Through digital platforms, mobile banking, and innovative payment solutions, Fintech can empower unbanked individuals, including those from lower-income households, marginalized communities, and individuals with limited access to physical banking infrastructure. By promoting financial inclusion, Fintech not only helps individuals manage their finances more effectively but also can support economic growth, poverty reduction, and social progress, aligning with the broader objectives outlined in the SDGs.

### Fintech’s talent demand

The Fintech market is currently evolving and in its early stages, but it holds great potential. In 2022, the market size was approximately $245 billion, and experts predict that it will skyrocket to $1.5 trillion by 2030, exhibiting a remarkable compounded annual growth rate of 25%. This surge can be attributed to the ongoing transformation of the traditional financial services value chain.

Interestingly, the demand for Fintech professionals has surged over the past few years. Since 2019, the number of job postings for Fintech-related roles has doubled, leading to a fierce increase of around 40% in the number of employers vying for top talent. This heightened demand and competition present a significant opportunity for higher educational institutions to meet the needs of these employers by producing the sought-after Fintech experts.

Higher education and industry need to collaborate to both develop and deliver new talent required in this nascent Fintech sector. Let’s provide an example of Massachusetts, our location that is likely replicated in many regions throughout the world. According to the Massachusetts High Technology Council (MHTC), when it comes to the number and concentration of Fintech job postings, California and New York take the lead. However, Massachusetts has experienced faster growth in Fintech postings compared to the rest of the United States. Over the past three years, the growth rate in Massachusetts was 30% per annum, surpassing its leading competitors. The MHTC further reveals that approximately 300 new companies have been actively searching for Fintech-related talent since 2019. These companies can be broadly categorized into two types: large banks aiming to develop Fintech capabilities and startups or new entrants in the industry.

A recent study conducted by the Pioneer Institute, a research think-tank, revealed a concerning gap between the number of graduates being produced by Massachusetts’s higher education institutions and the demands of the local Fintech industry. Surprisingly, even top-tier, graduate-level business schools have been slow to incorporate courses that bridge the gap between finance and technology (Barrett, 2018).^11^ In light of this dilemma, the Pioneer Institute raises an important question: if leading business schools are struggling to address this issue, where can Massachusetts’ public universities find inspiration to create a thriving and capable Fintech workforce? We will discuss this next in our transdisciplinary approach at WPI.

### A transdiscipliniary approach to foster transformative Fintech education

#### Dr. Joseph Sarkis: Transdisciplinary cooperation in Fintech education fosters professionals who possess a comprehensive understanding of various fields and can address real-world challenges

Cooperation with industry, government, and the broader community is also necessary to bridge the gap between scholarly endeavors and complex employer needs, from profit-making to responsible community engagement. From a research and skillset perspective, the private, public, and academic sectors need to be more creative with Fintech. It is not only a means for capital flows but also a tool for financial literacy in vulnerable communities. Developing appropriate skillsets aligned with emerging technologies is crucial for economic competitiveness and innovation (Spotti & Windelband, 2021).^12^

Furthermore, interdisciplinary Fintech programs contribute to economic development by producing graduates with the skills and knowledge to drive technological advancements in the financial sector. As Fintech continues to reshape traditional financial services, there is an increasing demand for individuals who can leverage technology to create new financial products and enhance efficiency. These programs have a positive impact on social welfare by democratizing access to financial services and addressing societal challenges such as financial inclusion and poverty alleviation. However, it is important to note that although a majority of Fintech job postings may be from the traditional financial sector, unsurprisingly a growing number of postings are from startups. We also believe non-profits and governmental jobs will also be seeing greater career opportunities for Fintech.

### WPI Fintech education context and Scale

#### *Dr*. *Debora Jackson (Dean of the WPI Business School)*: *WBS’ distinctive competence in its cross-disciplinary experiential learning*, *Major Qualifying Projects (MQP) (administered by our Fintech MQP*, *FinTech Project Center)*, *positions our students and faculty for the interdisciplinary requirements of new Fintech programs*

WPI, known globally for interdisciplinary project-based learning, was recently awarded the 2023 Institute of International Education Andrew Heiskell Award in the field of student mobility and exchange. The award recognizes WPI’s continuous endeavors to enhance its acclaimed Global Projects Program, which was established in 1974. Serving as an integral part of the WPI curriculum, the Global Projects Program offers students from all disciplines the opportunity to engage in intricate real-world challenges through interdisciplinary teamwork. We believe that this platform provides a basis for the development and advancement of programs in Fintech. At the WPI Business School (WBS), we believe that through collaboration among finance, computer science, engineering, and other relevant disciplines, higher education institutions can nurture a talent pool that meets industry demands and stimulates economic growth. Such Fintech initiatives facilitate knowledge exchange and research collaboration, leading to holistic solutions for complex financial and societal issues.

Moreover, WBS believes that the true achievements of Fintech should be led by the pursuit of social good, an example of a paradigm shift, in which we build an inclusive image for our community globally, focusing on the benefits that Fintech can bring to vulnerable populations, the disenfranchised, and the ostracized. In this regard, we have established partnerships with FLAME University in Pune, India and the Nigerian University of Technology and Management in Lagos, Nigeria with additional international partnerships in progress, fostering global collaboration. Our Fintech project centers implement Fintech knowledge in practical applications, such as using data analytics, the internet of things, and blockchain technology to address social good concerns for smaller businesses.

Research at WPI also demonstrates the interdisciplinary nature of Fintech. For instance, our research highly encourages transdisciplinary teams comprising blockchain technology experts, materials scientists, sustainability experts, and supply chain researchers to collaborate and address safety concerns in a circular economy. This research explores how incentives, enabled by Fintech, can be used to address both social and environmental challenges.

Our MQP project center administers several interdisciplinary projects each year that bring together students from various disciplines such as business, Fintech, computer science, mathematical sciences, data science, and more. In these projects, teams analyze business requirements and design, develop, and implement real-world Fintech solutions for several leading organizations across the Fintech ecosystem such as Angelo Gordon, Citizens, Fidelity Investments, State Street, Vestigo Ventures, BNP Paribas, Woo Sox, and several Fintech startups. Additionally, our FABLab for Social Good brings a number of projects to the project center each year. This interdisciplinary competence that we developed via our MQP project center prepares us well to organize our students into these multidisciplinary Fintech agile teams.

### WPI’S interdisciplinary Fintech approach: Q & A

We now feature several questions and answers with key members of the interdisciplinary Fintech program at WBS.

#### What was the motivation to launch your interdisciplinary research program and what are the main challenges you have faced so far? What are the key factors that stimulate interdisciplinary research?

Fintech is transforming the delivery of financial services, creating new business models amid changing customer expectations. However, global and social challenges need to be addressed. Consequently, there is a growing demand for professionals (and researchers) with the technical and business skills required to navigate this rapidly evolving landscape, where social good and broader social impacts are central to our work.

The motivation to launch a Fintech interdisciplinary degree and a Fintech research center (FABLab) for social good at WBS directly relates to its mission, which reads:

The WPI Business School develops adaptive leaders who create sustainable solutions, deliver globally responsible impact, and conduct transformative research at the intersection of business, technology, and people.

However, a major challenge that we faced in developing our programs involved keeping pace with not only a fast-changing industry (ensuring that the curriculum is relevant and up-to-date) but also the shifting social obligations landscape. Beyond environmental and economic sustainability, there are concerns and developments in equity, inclusion, and addressing various social ills. Constant effort would be required to remain informed of the latest developments in a field that has not typically had the willingness to adapt to these emerging social challenges.

Looking to the future, key challenges include:

• maintaining innovation that is practically feasible,
• encouraging cross-disciplinary collaboration among a diverse set of faculty members and researchers who have their own specialty areas, and
• ensuring that graduates are well prepared for the evolving Fintech landscape, well beyond bits, bytes, and dollars.

The key factors that stimulate interdisciplinary research include fostering a culture of collaboration and encouraging the exchange of ideas among researchers from different disciplines. We developed a program at WBS as a collaborative effort between our Global School and The School of Arts and Sciences: Computer Science, Data Science, and Mathematics departments. Our Fintech research center (FABLab) for social good promotes interdisciplinary research by organizing seminars, workshops, and conferences that bring together experts from various fields to discuss current challenges and opportunities in Fintech, artificial intelligence, and blockchain technology for social good. This is the focus of our WPI/Future Finance and Economics Association conference on “Fintech for inclusivity, growth, and the future.”

#### How do you prepare your students to communicate in interdisciplinary teams? How do you prepare the staff to teach, interact, and solve conflicts in interdisciplinary settings?

Strategies we have found to be successful over the years include:

1. Students are taught the importance of clear communication and active listening skills and are trained to communicate complex concepts in a way that is easily understood by collaborators from different backgrounds.
2. Even before the MQP, students at WPI are required to take a three-course equivalent (one full term) on an Interactive Qualifying Project, which is meant to address issues related to technology and society. In these projects, students from multiple disciplines are required to address a project by learning interdisciplinary team skills, while learning and applying skills and knowledge from their majors and other disciplines. The projects typically have strong social and sustainable elements.
3. Students are organized into groups with individuals from different backgrounds. This supports appreciation of diverse perspectives.
4. Students are trained to implement the Agile Scrum methodology in their development projects. This skillset is needed to be able to effectively meet goals within rapidly changing social, business, and technological contexts.
5. The teams also had access to the WPI SWEET Center. Teams that experienced difficulties with group dynamics were encouraged to contact the SWEET Center for individual or team consultations. Individual and team consultations are offered by SWEET fellows, who are WPI students, staff, faculty, and alumni with extensive project and teamwork experience, and additional training from WPI experts on effective and equitable teamwork. All SWEET Center offerings are available free of charge to WPI undergraduate and graduate students.

For our faculty:

1. The MQP project center fosters collaboration among faculty members from several disciplines. For instance, in the MQP center, faculty members work together with students in diverse multidisciplinary teams on projects or research initiatives.
2. This collaborative faculty effort across WPI majors helps students develop leadership skills, communication, capabilities, competencies, and team collaboration to deliver Fintech/finance industry solutions to real customers and institutions.
3. Interaction can also occur through faculty joint teaching in some courses and jointly guiding research for graduate students. For example, faculty are involved in funded programs that support computer science, data science, mathematics, physics, business, and social sciences for robotics and circular economy. Financial issues and technology concerns arise in each of these areas.

#### What specific methodological challenges can be faced by interdisciplinary researchers and how do you prepare your (students/researchers) for them?

Our experiential learning in the MQP project center and joint faculty research clarifies that research on interdisciplinary programs, such as Fintech for social good can present specific methodological challenges. Some of the challenges that we prepare our students to navigate include:
1. **Integration of multiple disciplines**: Faculty and students must find ways to bridge the gaps between different fields and develop a shared language and understanding of key concepts and terminology.
2. **Managing complexity**: Fintech, especially its expansion into social good and sustainability research, is a complex and rapidly evolving field, which can be challenging to navigate. Faculty and students need to be able to manage complexity and uncertainty and develop methods for synthesizing and analyzing large amounts of data.
3. **Identifying appropriate research methods**: Interdisciplinary research requires multiple research methods, which can be difficult to navigate. Faculty and students must be able to identify appropriate methods and techniques from different fields and integrate them effectively. These methods range from qualitative evaluation, formal analytical modeling, and programming to empirical statistical modeling and quantitative analysis. There also needs to be trust that the proposed methodologies are appropriate for answering these questions. Challenges include convincing individuals and team members that the most appropriate methodology is selected.

#### What is your recipe for governance of interdisciplinary projects (e.g., acquiring funding, project planning, and management)? How do you prepare your (students/researchers) to participate in these challenges?

The following points comprise a recipe for effective governance, which we developed over the years from similar interdisciplinary endeavors:

1. **Identify project goals**: We require students and researchers to clearly define the goals and objectives of the project. This will help guide project planning and ensure that all stakeholders are aligned.
2. **Establish a project team**: The MQP center and FABLab for social good works should organize well-balanced, cross-functional teams with the necessary skills and expertise to execute the project. Teams should include individuals with various skill sets and competencies to provide diverse perspectives and insights. For example, we have management, finance, and computer science scholars and students attempting to address the question of how blockchain can be used to manage sensor data that may have social good implications, while developing a business model for this technology and the company partner. Different skill sets, including finance, sustainability, supply chain, management, computer science, and entrepreneurship faculty skills, need to collaborate with students across disciplines to effectively address just one small startup’s needs.
3. **Develop a project plan**: Develop a detailed project plan that outlines the project’s activities, timelines, and deliverables. The plan should be regularly reviewed and updated to ensure that the project remains on track.
4. **Implement project management processes**: Establish project management processes such as regular status meetings, progress reports, and risk management plans. This will help ensure that the project stays on track and that any issues are identified and addressed promptly.
5. **Funding**: As we identify funding opportunities for FABLab, such as grants or industry partnerships, we ensure that proposals are developed by a cross-functional team of scholars.

#### When publishing/disseminating interdisciplinary work, what are some strategies that you applied to reach a broader audience beyond specific disciplines? How are these reflected in students’ training?

1. **Use accessible language**: When writing about interdisciplinary work, it is important to use accessible language that is easy for the layperson to understand.
2. **Collaborate with non-academic partners**: Collaborating with non-academic partners, such as industry or government, can help increase the reach of interdisciplinary work to become transdisciplinary. This can also help ensure that the research is relevant to real-world problems. For example, we blogged about a Massachusetts Technology Leadership group that allowed us to reach out to various professional and academic communities related to our Fintech for social good research and outreach efforts. Our FABLab for social good research includes WorcLAB, a local entrepreneurial incubator located in the city center and close to underrepresented and vulnerable communities. This partnership allows us to reach out to communities that Fintech and blockchain researchers typically do not. We have also been working closely with state and local city officials on topics related to smart cities and their relationship to Fintech. One project that we are working on, for example, is something in which our state legislatures have an interest in—blockchain applications for the government. Currently, we have Ph.D. students conducting a structured literature review for application and use cases across government agencies that can inform not only legislation but also actual application in government settings (which include a strong foundation of Fintech).
3. **Engage in public outreach**: Engaging in public outreach, such as presenting research findings at conferences or authoring articles for popular media outlets, can help increase the visibility of interdisciplinary work. This is a common Fintech practice in Massachusetts professional groups. We are involved in the Boston Blockchain Association workshops that include our faculty, students, governmental officials, industry, and incubators.
4. **Use social media**: Social media can be a powerful tool for reaching a broader audience. Researchers can use social media platforms, to share their work and engage with a wider community and publicize our activities and publications. Our blogs and meetings with descriptions of the outcomes appear across these different social media platforms. When an article, especially one that is open access, is available, we do our best to individually or as a school support its marketing.

#### What are the future outlooks of interdisciplinary research at your institution? What tips would you give to anyone considering undertaking interdisciplinary work?

The future outlook for interdisciplinary research at WPI is positive, as we see continued growth and interest in interdisciplinary research, particularly in the field of Fintech. We anticipate that interdisciplinary research will become increasingly important in addressing complex problems and driving innovation. For example, WBS’ departments are not physically separated, rather, faculty members are physically located next to each other. In one hallway, we have marketing, information systems, professor of Fintech practice, sustainability, and supply chain, and a finance professor right next to each other. One piece of advice is to provide easy access to each other and physically mix disciplines (at least from a business perspective). The interactions become organic and ideas continue to flow as the faculty can communicate very easily.

For others considering undertaking interdisciplinary work, we would offer the following tips:

1. **Start with a clear problem statement:** Interdisciplinary research works best when there is a clear problem or challenge to be addressed. Start by defining the problem and identifying the expertise required to solve it. We have many potential topics for consideration. The problem is sometimes very complex and requires several insights. For example, environmental, social, and governance (ESG) investing is now an important dimension of Fintech and sustainability. Faculty members have access to each other to share ideas on the limitations and concerns of ESG. One such example is the large number of ESG indicators. The finance, operations analytics, and information systems faculties have different methodologies and perspectives on this issue. Sometimes they would need to figure out whether tools, terminology, or the problem can be addressed simultaneously. They can then narrow down the concerns (which are usually complex) into significant questions that can be jointly discussed and addressed.
2. **Build a diverse team of faculty experts:** Assemble a team of faculty experts with diverse backgrounds, skills, and expertise. This will help ensure that all perspectives are represented and the team can work effectively across disciplinary boundaries. Over the past few years (including during COVID), we have been introducing and bringing faculty members in who are researchers in Fintech and blockchain. We also encouraged them to reach out to current faculty members in other disciplines who they can complement. These collaborations happen sometimes through introductions and sometimes through informal gatherings.
3. **Develop shared language and understanding of key concepts across disciplines**: This will help ensure effective communication and collaboration. We have held meetings with different constituencies and stakeholders, where discussions, readings, and presentations increase shared understanding. We have Ph.D. student seminars, a faculty program called TREES, and various formal and informal meetings in which ideas and terminology are presented. Faculty and students are encouraged to ask questions and engage in dialog. Formal and informal mixtures exist. Quite often, faculty members share with each other and students, and sometimes even administrators, articles and electronic sites regarding various topics. This allows for not only updating each other on emergent issues but also forming a common understanding and language.
4. Challenges that may be encountered in interdisciplinary research include:
  • **Difficulty in finding common ground**: Researchers from different disciplines may have different priorities and goals, making it challenging to find common ground and agree on a research agenda.
  • **Lack of funding opportunities:** Funding opportunities for interdisciplinary research may be limited, making it challenging to secure funding for projects involving multiple disciplines. This is especially true when business and management scholars seek funding from science and engineering research programs, such as the National Science Foundation in the U.S. Typically, they require scientific breakthroughs, and a management or business scholar will have a difficult time without interdisciplinary research with various science and engineering scholars. Some government programs support research in business and entrepreneurship; however, they typically have to be tied to economic development goals.
  • **Resistance to change**: Interdisciplinary research may challenge traditional disciplinary boundaries and ways of thinking, which researchers or institutions might resist.
